# Microarray for Identification of the Chiropteran Host Species of Rabies Virus in Canada

**DOI:** 10.3390/microarrays2020153

**Published:** 2013-05-31

**Authors:** Oliver Lung, Susan Nadin-Davis, Mathew Fisher, Anthony Erickson, M. Kimberly Knowles, Tara Furukawa-Stoffer, Aruna Ambagala

**Affiliations:** 1 Canadian Food Inspection Agency, National Centres for Animal Disease, Lethbridge Laboratory, P.O. Box 640, Lethbridge, AB T1J 3Z4, Canada; E-Mails: Mathew.Fisher@inspection.gc.ca (M.F.); Anthony.Erickson@inspection.gc.ca (A.E.); Tara.Furukawa-Stoffer@inspection.gc.ca (T.F.-S.); Aruna.Ambagala@inspection.gc.ca (A.A.); 2 Canadian Food Inspection Agency, Ottawa Laboratory Fallowfield, 3851 Fallowfield Road, P.O. Box 11300, Ottawa, ON K2H 8P9, Canada; E-Mails: Susan.Nadin-Davis@inspection.gc.ca (S.N.-D.); Kim.Knowles@inspection.gc.ca (M.K.K.)

**Keywords:** bats, microarray, rabies, Chiroptera, COI

## Abstract

Species identification through genetic barcoding can augment traditional taxonomic methods, which rely on morphological features of the specimen. Such approaches are especially valuable when specimens are in poor condition or comprise very limited material, a situation that often applies to chiropteran (bat) specimens submitted to the Canadian Food Inspection Agency for rabies diagnosis. Coupled with phenotypic plasticity of many species and inconclusive taxonomic keys, species identification using only morphological traits can be challenging. In this study, a microarray assay with associated PCR of the mitochondrial cytochrome *c* oxidase subunit I (COI) gene was developed for differentiation of 14 bat species submitted to the Canadian Food Inspection Agency from 1985–2012 for rabies diagnosis. The assay was validated with a reference collection of DNA from 153 field samples, all of which had been barcoded previously. The COI gene from 152 samples which included multiple specimens of each target species were successfully amplified by PCR and accurately identified by the microarray. One sample that was severely decomposed failed to amplify with PCR primers developed in this study, but amplified weakly after switching to alternate primers and was accurately typed by the microarray. Thus, the chiropteran microarray was able to accurately differentiate between the 14 species of Canadian bats targeted. This PCR and microarray assay would allow unequivocal identification to species of most, if not all, bat specimens submitted for rabies diagnosis in Canada.

## 1. Introduction

The emergence of genetic tools to explore species differences has had a tremendous impact on our knowledge of global biodiversity and has in some instances provided greater insight into higher order taxonomic relationships [1]. In particular, due to the mitochondrial genome’s limited intra-specific variation but significant inter-specific divergence, partial nucleotide sequencing (barcoding) of certain mitochondrial loci for the purpose of species discrimination has gained widespread acceptance [2,3]. Two mitochondrial loci, cyt b (cytochrome b) and COI (cytochrome *c* oxidase subunit I), have gained particular prominence in this role [2,4]. Based on the pioneering work of Hebert and colleagues [5,6], the international barcode of life (IBOL) consortium, which seeks to generate millions of DNA barcodes for thousands of species world-wide, has adopted COI barcodes as the standard for studies on biodiversity throughout the animal kingdom [7]. 

Barcoding of one mammalian order, the Chiroptera, has increased our knowledge of diversity within this group significantly [8,9]. With approximately 1,240 species worldwide, the chiroptera is the second largest order of mammals, comprising more than 20% of the species in the class Mammalia [10,11,12]. Moreover bats are now recognized as important hosts involved in the emergence and spread of animal and zoonotic viruses belonging to a wide range of families including the *Rhabdoviridae* (e.g., Rabies virus), *Paramyxoviridae* (e.g., Nipah and Hendra virus), *Coronaviridae* (e.g., SARS-CoV), and *Filoviridae* (e.g., Ebola and Marburg virus) [13,14,15,16]. Indeed many bat species figure prominently as the reservoirs for members of the *Lyssavirus* genus, family *Rhabdoviridae*, a group of negative strand RNA-viruses that are the causative agents of the zoonotic disease rabies [15,17]. 

*Rabies virus* (RV) is the type species of this genus and the only *Lyssavirus* known to circulate in the Americas [17]. Throughout the continent, including Canada, multiple RV strains are maintained by specific wildlife hosts, of both carnivora and chiroptera orders, within discrete but overlapping geographic areas and there is a need for tools that discriminate both host species and viral strains [18,19,20]. Currently, hundreds of confirmed rabid bats are reported annually in North America [17] and the persistence of RV in a broad range of native bat species continues to be a public health hazard due to the high fatality rate of this disease and frequent failure of the public to associate bat contact and even superficial bites with potential rabies exposure [21,22]. While timely post-exposure or pre-exposure vaccination can prevent rabies in humans and other mammals, after onset of clinical disease the prognosis is extremely poor with almost 100% mortality [23]. Additionally, the persistence of RV in chiropteran hosts provides a source of potential cross-species infection for domestic animals and can, on rare occasions, result in the establishment of bat RV variants in terrestrial wildlife [24], events which have the potential to undermine rabies control activities in non-flying RV reservoir species. Improved knowledge of the epidemiology and geographical distribution of bat-associated RVs, including the species responsible for disease maintenance, would facilitate better risk management of this disease and limit its impact on the health of humans and domestic animals. 

In Canada, rabies diagnosis is routinely performed by the Rabies Centre of Expertise of the Canadian Food Inspection Agency (CFIA). Large numbers of bats are submitted annually and species determination is currently based on traditional taxonomic methods in which the specimen is visually examined for anatomical features consistent with each species [25,26,27]. Although 17 species of bats are considered indigenous to Canada [28] three species, the spotted bat (*Euderma maculatum*), eastern small-footed bat (*Myotis leibii*), and the western small-footed bat (*Myotis ciliolabrum*) rarely come in contact with humans due to their scarcity and/or their very limited ranges within Canada. As a result they are virtually never submitted for rabies testing. The pallid bat (*Antrozous pallidus*) is also uncommon and very infrequently submitted so the vast majority of submitted specimens represent just 13 species. The bat samples received are often in poor condition due to decomposition or injury, or in some cases are incomplete (e.g., head only), thereby making species identification difficult or even impossible. In addition, some species (particularly of the *Myotis* genus) share highly similar physical features which further challenge species identification of these samples. To address these issues a recent study, which generated barcodes for 260 bat specimens representing 13 Canadian bat species, proposed the use of COI barcoding methods to improve species identification of submitted specimens [29]. Similar strategies involving sequence determination of other mitochondrial loci have been applied to confirm the nature of rabies reservoir species in Latin America [30], and to expand knowledge of the epidemiology of bat lyssaviruses in Europe [31]. However, to facilitate routine use of such an approach in a diagnostic setting it was determined that a highly automated method which could be applied by analysts not expert in complex molecular biology techniques would be highly valuable.

Given the capability of DNA microarray technology to undertake massively parallel analyses in widely diverse biological systems [32,33], a project to develop a rapid and robust method for Canadian bat species identification which combines microarray and bat barcoding technologies has been initiated. A number of studies have combined microarray and DNA barcoding methods for identification of mammalian [34,35], insect [36], fish [37], bird [38], and fungal [39] species. Using these same principles this report describes the development of a universal PCR for amplification of the COI gene and the development of a microarray for differentiation of 14 Canadian bat species that represent virtually all specimens submitted to the CFIA for rabies diagnosis for the past 20 years. This study demonstrates the feasibility of this approach as a first step in the development of a novel user-friendly molecular method for bat species identification, a tool which will be invaluable in supporting bat rabies surveillance in Canada. 

## 2. Experimental Section

### 2.1. Bat Specimens

Specimens used in this study were randomly selected from frozen archived bat tissues submitted to CFIA laboratories located in Ottawa, Ontario and Lethbridge, Alberta for rabies diagnosis between the years 1985–2012 (Table 1). Submitted specimens are routinely examined upon arrival at the laboratory by diagnostic staff and assigned a species designation based on specific morphological keys. All of the samples included in this study had also been assigned to species based on COI barcoding as described previously [29].

**Table 1:** Bat species investigated in study.

Species	Abbreviation	Scientific Name	Numbers Tested
Little brown bat	LBB	*Myotis lucifugus*	24
Northern long-eared bat	NLB	*Myotis septentrionalis*	13
California bat	CLB	*Myotis californicus*	7
Long-legged bat	LLB	*Myotis volans*	5
Western long-eared bat	LEB	*Myotis evotis*	12
Yuma bat	YUB	*Myotis yumanensis*	8
Keen’s bat	KEB	*Myotis keenii*	14
Big brown bat	BBB	*Eptesicus fuscus*	25
Townsend’s big-eared bat	WEB	*Corynorhinus townsendii*	9
Hoary bat	HRB	*Lasiurus cinereus*	10
Eastern red bat	REB	*Lasiurus borealis*	9
Silver-haired bat	SHB	*Lasionycteris noctivagans*	12
Tricolored bat *	EPB	*Perimyotis subflavus*	3
Pallid bat	PAB	*Antrozous pallidus*	2
		*Total*	153

* Formerly referred to as the Eastern pipistrelle bat (*Pipistrellus subflavus*).

### 2.2. Primer and Probe Design

A custom database was initially developed that contained 365 American and 685 Canadian bat COI gene sequences obtained from NCBI, Barcode of Life Data Systems (BOLD) [7] and CFIA ([29] and Nadin-Davis, unpublished results). Due to the presence of gaps in the sequence information, several sets of PCR primers were designed to sequence the COI gene of the targeted species. Sequence across the entire or most of the COI gene was obtained for all targeted species. Multiple sequence alignments were performed using Clone Manager Professional Version 9 (Science & Educational Software) and used to identify highly conserved regions which were then used to design PCR primers for amplification of the COI gene of the targeted species. 

For probe design, COI sequences generated from 13 Canadian bat species for which specimens were initially available were aligned using ClustalXv2 or ClustalXv1.83 [40,41] and then reviewed manually to identify regions that were conserved within a species but which contained multiple substitutions when compared with all other species. For each species at least two discrete regions of the gene were identified and probes targeting these sequences were designed and optimized using the OligoAnalyzer^®^ tool [42]. Once a sequence for the previously unavailable pallid bat COI gene became available, this information was included in subsequent alignments and a pallid bat probe was designed using the program AlleleID (Premier Biosoft International). This probe was subsequently examined for specificity by BLAST analysis against full length COI sequences of the targeted bat species. 

### 2.3. DNA Extraction

Approximately 50 mg of brain, lung, spleen, or hair bud were taken from frozen bat specimens for DNA extraction using either of two methods. In most cases the sample was ground in a hexadecyl trimethyl ammonium bromide (Sigma-Aldrich, Oakville, ON, Canada) solution, subjected to phase separation after addition of chloroform and the aqueous phase was then precipitated with alcohol as described previously [43]. The final dried DNA pellet was dissolved in TE buffer (10 mM Tris-HCl, pH 8.0, 0.1 mM EDTA) and stored at −20 °C. Alternatively, some samples were extracted using a DNeasy Blood and Tissue Kit (Qiagen, Toronto, ON, Canada) as per the manufacturer’s recommendations. Nucleic acid concentration was quantified by UV absorbance at 260 nm on a NanoDrop 8000 (Thermofisher Scientific, Toronto, ON, Canada) or Nanovue instrument (GE Healthcare, Mississauga, ON, Canada).

### 2.4. Slot Blot Analysis

Slot blot analysis was used in the preliminary screening of capture probes. COI PCR products, amplified as described previously [29] and purified using Wizard^®^ PCR Preps DNA Purification System (Promega, Madison, WI, USA) according to the manufacturer’s instructions, were normalized to similar concentrations based on their intensity upon gel electrophoresis. These amplicons were then blotted on to Hybond nylon membranes (GE Healthcare) using a Minifold II slot blot system (Schleicher & Schuell, Keene, NH, USA) followed by fixation using UV cross-linking. Oligonucleotide probes were labelled by 3′ tailing with digoxigenin (DIG) using DIG-11-dUTP and terminal transferase (Roche Applied Science, Laval, QC, Canada) as recommended by the manufacturer. The slot blots were incubated in pre-hybridization buffer (5× SSC, 1% casein, 0.1% N-lauryl sarcosine, 0.02% SDS) for 1 h and the DIG-labelled probes were then added and allowed to hybridize overnight at either 37 °C or 42 °C. After hybridization, blots were washed sequentially in 0.1% SDS containing 2× SSC, 1× SSC and 0.5× SSC at the hybridization temperature, and once in Buffer 1 (0.1 M Tris-HCl, 0.15 M NaCl, pH 7.5) at room temperature. All remaining manipulations were conducted at room temperature. The blots were then blocked for 1 h in blocking buffer (Buffer 1 containing 1% casein) prior to application of anti-DIG alkaline phosphatase (AP) conjugate (Roche Applied Science) diluted 1:2,000 in blocking buffer for 30–60 min. Unbound conjugate was removed by washing twice in buffer 1 for 15 min each time, and once in Buffer 3 (0.1 M Tris-HCl, 0.1 M NaCl, 50 mM MgCl_2_, pH 9.6) for 10 min. Fresh Buffer 3 containing NBT/BCIP substrate (Roche Applied Science) was applied and colour development proceeded in the dark for 1–2 h until it was terminated by washing in water. Blots were subsequently imaged using a Gel Doc system (Bio-Rad Laboratories, Mississauga, ON, Canada).

### 2.5. PCR Amplification for Microarray Analysis

Approximately 200 ng of DNA was used for PCR amplification of the COI gene. All PCR amplifications were performed in 50 μL reactions using Platinum^®^ *Taq* DNA Polymerase (Life Technologies, Burlington, ON, Canada). PCR consisted of an initial denaturation step at 94 °C for 3 min, followed by 40 cycles of 94 °C for 45 s, 45 °C for 45 s, 72 °C for 1 min, and a final extension step of 72 °C for 10 min. The 50 μL reaction mixture consisted of 2 μL of DNA template, 0.2 μL of enzyme mix in 5 µL 10× reaction buffer, 1.5 μL of 50 mM MgCl_2_, 1 μL of 10 mM dNTP mixture, and 1 μM of each primer. The COI primers used for PCR amplification were AECOX-1TAG FWD-3.0 and AECOX-R1A (details are provided in Section 3.1). Alternate primers used to amplify a badly decomposed specimen were COX-F4 (5′-TCAACCAATCAYAAAGAYATTGGTAC-3′) and COX-R4 (5′-GTGAAYATATGGTGGGCTCATACGAT-3′). Following PCR, amplicons were visualized either on the QIAxcel instrument (Qiagen) or on a 1% agarose gel. The remainder of the amplified products were fluorescently labelled using the ARES Alexa Fluor 647 Labelling Kit (Life Technologies) as previously described [44]. Briefly, following amplification, PCR products were hydrolyzed to remove RNA and then purified using DNA Clean and Concentrator spin columns (Zymo Research, Irvine, CA, USA). Aminoallyl dUTP was incorporated by random priming and the product was purified using spin columns, and labelled using Alexa Fluor 647 dye. The fluorescently labelled samples were purified using spin columns and stored at 4 °C until use.

### 2.6. Microarray

Capture probe printing, hybridization, washing and reporting were performed as previously described [44]. Briefly, probes in 15 μL Pronto!^TM^ Epoxide Spotting Solution (Corning, Tewksbury, MA, USA) were spotted in triplicate on Epoxide Slides (Corning) at 60% humidity using the VersArray ChipWriter ^TM^ Pro printer (Bio-Rad) and SMP3 pins (ArrayIt, Sunnyvale, CA, USA). Following printing, the humidity was increased to 70% overnight and then gradually reduced to ambient humidity the following day. Printed slides were stored in a desiccating chamber until use. Printed slides were blocked by incubation in pre-hybridization buffer (5× SSC, 0.1% SDS and 0.1 mg/mL BSA) for 45 min at 42 °C. Slides were then washed three times in 0.1× SSC and once in Milli-Q water before being dried using a slide centrifuge (ArrayIt), and loaded onto a 24-well hybridization cassette (ArrayIt). Twenty-one microlitres of labelled amplicon was mixed with an equal volume of hybridization buffer (20% formamide, 10× SSC, 0.2% SDS, and 0.2 mg/mL salmon sperm DNA), denatured at 95 °C for 5 min and then cooled to room temperature. The labelled amplicon and hybridization buffer mixture was loaded into individual wells of the hybridization cassette, which was then sealed and incubated overnight at 42 °C. The following day, hybridized slides were washed once in 2× SSC, 0.1% SDS at 42 °C, twice in 1× SSC and twice in 0.1× SSC at room temperature. Finally, slides were dried by centrifugation and scanned using the GenePix 4000B scanner (Molecular Devices, Sunnyvale, CA, USA). Image analysis was performed using GenePix Pro software version 5.0 (Molecular Devices). The reactivity of each sample to the capture probes was represented using the average median fluorescent intensity (MFI) of the replicates. Probe reactivity was considered positive if it had an MFI greater than the mean MFI of the whole probe set plus two times the standard deviation. This cut-off value was calculated individually for each sample.

## 3. Results and Discussion

### 3.1. Development of a Universal PCR Primer Pair

The 14 indigenous Canadian bat species targeted by this study are listed in Table 1. A previous study of the variation of the COI gene in 13 Canadian bat species required the use of four separate PCR reactions for successful amplification of the COI gene from all the species [29]. Refinement of this PCR to facilitate target amplification by a single universal primer set was a prerequisite for efficient microarray analysis. Accordingly, an alignment of all available 685 Canadian bat COI gene sequences was examined to assist with primer design. This revealed gaps in the required sequence information. Thus multiple sets of PCR primers flanking the COI gene were designed and used to amplify the entire COI gene of single specimens for 13 of the 14 targeted Canadian bat species, and the majority of the COI gene of the remaining species (tricolored bat). The information obtained from sequencing the COI amplicons allowed design of a single primer pair for partial amplification of the COI gene of all 14 species. These COI primers were AECOX-1TAG FWD-3.0 (5′-TAGRTTTACAGYCTAATRCCTACTC-3′) and AECOX-R1A (5′-ACYTCAGGRTGRCCAAARAATCA-3′).

### 3.2. PCR for Partial Amplification of the Cytochrome c Oxidase Subunit I (COI) Gene of Canadian Bats

**Figure 1:**
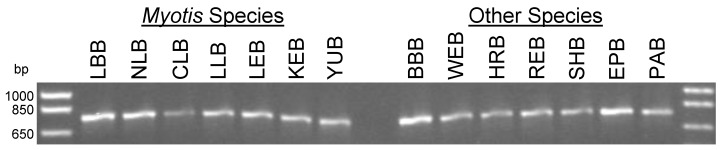
Gel image of PCR amplified material from representative samples of 14 Canadian bat species targeted in this study. A primer pair developed in this study was used for the PCR and the targeted cytochrome *c* oxidase subunit I (COI) regions of all 14 bat species were successfully amplified.

The PCR developed in this study generated amplicons of 766 bp (Figure 1). The PCR was validated using DNA extracted from 153 Canadian field specimens of the 14 bat species. While many of the DNA samples included in this study were of poor quality based on analysis by gel electrophoresis (data not shown), all but one specimen were successfully amplified, including multiple samples from each target species. Thus these results indicate that for the vast majority of samples that were encountered, the universal PCR primers designed in this study can successfully replace the four separate PCRs [29] previously used to amplify the partial COI gene of the targeted bat species. The one sample that failed to amplify was badly decomposed and a weak product was only generated after switching to alternate primers that produced a slightly different amplicon of approximately the same size (data not shown). As shown in the following sections and Table 2, for the purpose of this application a sufficiently large COI sequence which encompasses significant genetic diversity to allow use of multiple capture probes is necessary for accurate species identification. However given the possibility that other suboptimal field specimens are likely to be encountered, reduction in amplicon size may improve PCR product yields as described in other studies which used smaller microcodes [45,46]. Thus amplification of this region of COI as two smaller multiplexed PCRs each <400 bp could be entertained. 

**Table 2:** Description of all oligonucleotides evaluated for their use as species-specific probes.

Name	Specific Target	Sequence (5′-3′)	Location ^1^
BB1 ^2^	BBB	CTGCCCTGAGTCTGCT	130–145
BB2 ^2^	BBB	GTGGACCTGACCATT	465–479
BB-E ^2^	BBB (eastern)	CTTTTATTCGGCGCTTGA	96–113
BB-W ^2^	BBB (western)	TTCTGTTCGGCGCCTGA	97–113
EP1	EPB	CGCACACGCCTTTG	215–228
EP2 ^2^	EPB	CTTTCTTTTACTCCTAGCAT	362–381
LAS	HRB/REB	TATTATTGGCATCWTC	370–385
HR1 ^2^	HRB	AGTACCACTGATGATTGG	284–301
HR2 ^2^	HRB	CCACCTGCCTTGTCC	555–569
RE1 ^2^	REB	TCTATAACGTTATCGTGAC	196–214
RE2 ^2^	REB	GGTACCCCTTATRATCG	284–300
SH1	SHB	GGGCTCTACTTGGAGAT	172–188
SH2 ^2^	SHB	CAACTGGTTAGTTCCTCTG	275–293
WE1 ^2^	WEB	TGATTAATCCCACTAATGAT	279–298
WE2 ^2^	WEB	CTTACATCTGGCTGG	485–497
CL1 ^2^	CLB	CTGGGAGACGATCAAATT	180–197
CL2 ^2^	CLB	TTTTATAGTTATGCCAATCAT	239–259
LB1 ^2^	LBB	CGCTGAGCTAGGTCAA	152–167
LB2 ^2^	LBB	TTTCTATTACTGCTGGC	363–379
LE1	LEB	TGACATAGCCTTTCC	308–322
LE2 ^2^	LEB	CTGTCTTACTCCTTCTC	613–629
LL1	LLB	TGTTGGGGGACGATCAGA	178–195
LL2 ^2^	LLB	TCTTCTCTCTGCACTTA	478–494
NL1	NLB	AACTGGGCCAGCCA	157–170
NL2 ^2^	NLB	ATTCGTTTGGTCCGT	587–601
NL3 ^2^	NLB	ATTCGTTTGGTCTGT	587–601
KE1 ^2^	KEB	TCATAGTTATGCCCATTA	241–258
KE2	KEB	TATGCCCATTATAATTGG	248–265
YU1	YUB	CCCTTTTAGGGGATG	175–189
YU2 ^2^	YUB	TATAGTAATGCCGATTATAATC	242–263
PA ^2^	PAB	TAATGTAATTGTCACAGCA	200–218

^1^ Location of targeted sequence within the 766 bp amplicon which corresponds to positions 5,307 to 6,072 of a *Lasiurus borealis* mitochondrial genome (Genbank Accession NC_016873); ^2^ Probes selected for the minimal microarray panel to differentiate 14 Canadian bat species.

### 3.3. Evaluation of Probes by Slot Blot Analysis

Thirty candidate species-specific oligonucleotide probes were designed based on previously published partial COI gene sequences [29]. This included a broadly reactive LAS probe designed to detect both species of the *Lasiurus* genus, the eastern red bat (REB) and hoary bat (HRB), included in this study. A total of four probes directed to the big brown bat (BBB) were designed; some of these were designed to differentiate between bats of the western population (restricted to British Columbia) and those of an eastern population that is distributed across the rest of the country (from Alberta to Eastern Canada) as described previously [47]. This collection of probes did not include a probe for the pallid bat (PAB), as information on this species was not available until later in the study when this probe was incorporated into subsequent microarray analysis.

**Figure 2:**
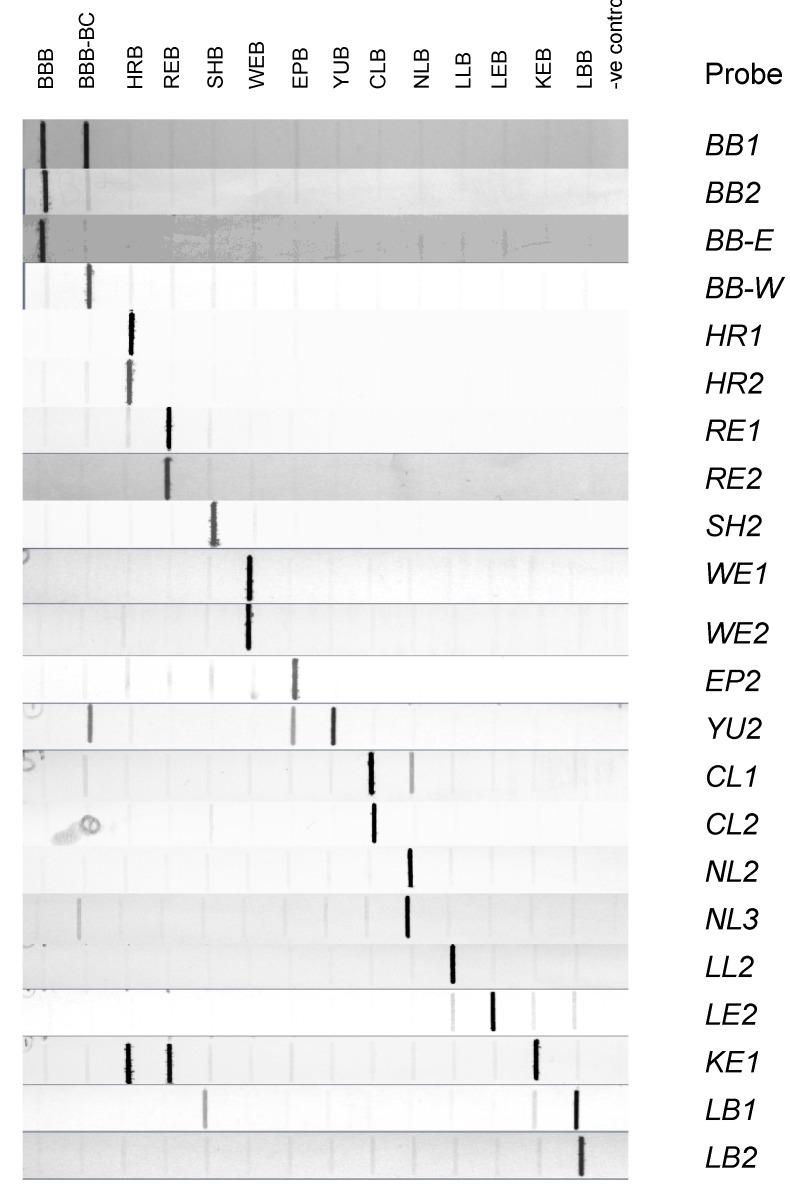
Composite diagram of 22 representative slot blots. Similar quantities of COI PCR product generated from 13 Canadian bat species were transferred to nylon membrane and each blot was hybridized at 37 °C with a probe as described. The bat species in which the PCR amplicon was derived and applied to each slot is indicated across the top, and the probes used for each blot are shown on the right hand side. Water was used in place of a PCR product for the negative (-ve) control. The probes included in this representation of the complete data were those chosen for the minimal microarray protocol with the exception of the *PA* probe that was not evaluated by slot blot analysis.

The reactivity and specificity of the 30 DIG-labelled probes on slot blots were first examined using COI PCR products generated from representative members of each of the initial 13 species. At a hybridization temperature of 37 °C, all probes exhibited strong binding to their targeted species and many showed either no or very weak cross reactivity with other species, as illustrated for selected probes in Figure 2. 

The *BB1* probe bound to both big brown bat (BBB) sub-types while *BB2* bound strongly only to the eastern BBB type. The *BB-E* and *BB-W* probes behaved as expected by detecting only the targeted sub-type. Probes that exhibited some moderate cross-reactivity included: *LB1*, which bound to the silver-haired bat (SHB) product and *CL1* which bound to the northern long-eared bat (NLB) product (Figure 2). The probes for Yuma bat (YUB) and Keen’s bat (KEB) were more problematic; *YU2* cross-reacted moderately with both the western-BBB and tricolored bat (EPB) samples (Figure 2) although this appeared to be substantially reduced when the hybridization temperature was raised to 42 °C (data not shown). Both probes for KEB exhibited significant binding to some of the other species; strong binding of the probe *KE1* to products of the *Lasiurus* bats (HRB and REB), as shown in Figure 2, was partly reduced by increasing the hybridization temperature to 42 °C but this did not entirely eliminate cross-reactivity with the HRB sample. However, overall these results were promising and more extensive evaluation of all probes was subsequently conducted in a microarray format. 

### 3.4. Evaluation and Validation of Probes by Microarray

**Figure 3:**
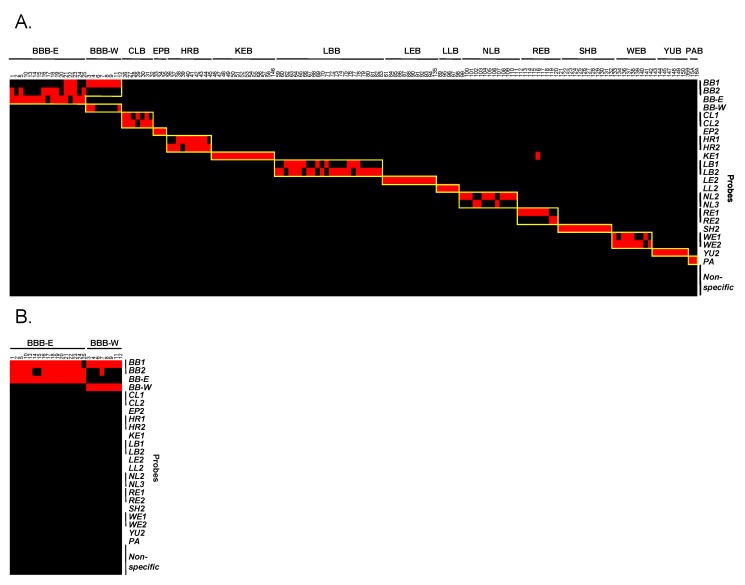
Summary heat maps of microarray results for field specimens. Specimen number and species are listed above the heat map while probes are listed to the right of the heat map. Positive reactions are indicated in red, and negative reactions in black. **Panel** **A**: Heat map of results for all 153 field specimens. A cutoff of two times the standard deviation of the fluorescent intensity for all probes plus the mean fluorescent intensity of all probes was selected for each sample for positivity. Specific probes for each sample are indicated by a yellow box. All specimens were accurately assigned. The outlying signal with the *KE1* probe is due to cross-reactivity with an eastern red bat (REB) specimen with a polymorphism which resulted in just a single mismatch to the Keen’s bat probe (*KE1*). **Panel** **B**: Heat map of microarray results for 25 big brown bat (BBB) field specimens within the panel. A cutoff of 0.25 times the standard deviation of the fluorescent intensity for all probes plus the mean fluorescent intensity of all probes was selected for each sample for positivity. All BBB specimens were accurately classified into western (British Columbia) and eastern (rest of Canada) populations.

All 30 candidate species-specific capture probes from the slot blot study and a new pallid bat probe were printed on microarray slides and screened for their utility in differentiation of the 14 targeted Canadian bat species. The probes were tested using DNA amplified from 153 field specimens. Each of the 14 bat species (Table 1) was represented by multiple specimens. Probes that showed significant cross-reactivity with heterologous species were eliminated. One or more capture probes were selected for each species and all 14 species were accurately differentiated using a set of 23 probes (Figure 3(A)). The sequences of all capture probes and their relative positions within the 766 bp amplicon are presented in Table 2; a subset was identified as a minimal discriminatory panel. 

For four of the 153 specimens tested, species assignments were inconsistent with the original species designation based on morphological criteria. DNA sequences of amplicons derived from discordant samples were consistent with species designation based on microarray results and with previous barcoding results (data not shown), suggesting errors in the initial phenotypic species designation. Species-specific probes for 13 of the 14 species in the final probe set were highly specific to the intended species and did not show cross reactivity with heterologous species. The Keen’s bat (KEB) probe (*KE1*) showed some cross reactivity with specimens from two heterologous species, but did not affect accurate species identification. 

All nine eastern red bat (REB) specimens tested were detected by the *RE* probes (*RE1* and *RE2*) as expected. However, one REB sample (#116) also showed strong reactivity with the *KE1* probe for Keen’s bat (P/N = 99.0) that was not observed with the other eight REB specimens (Figure 3(A)). Sequencing of the amplicon from sample #116 revealed that the *KE1* probe binding region had a single mismatch with the penultimate nucleotide of the *KE1* probe, while the amplicon from the other eight REB specimens had an additional mismatch with a nucleotide near the center of the *KE1* probe (Table 3). Only four out of 42 COI sequences from REB in the custom database had the same sequence as sample #116 in the *KE1* probe binding region while 38 out of 42 (90.5%) had two mismatches in the *KE1* binding region. The observation that the majority of the REB *CO1* sequences present in the database have two mismatches with the *KE1* probe suggest that most REB specimens will likely not react with this probe. The existence of a small number of REB sequences with polymorphisms that allows binding to the *KE1* probe indicates that samples which react with both *RE* and *KE1* probes should be categorized as an eastern red bat (REB). 

**Table 3:** Polymorphism observed in the *KE1* capture probe binding region of eastern red bats (REB).

Sample/Probe	Sequence (5′-3′)
REB #116	T**T**A TAG TTA TGC CCA TTA ^1^
REB #115	T**T**A TAG T**C**A TGC CCA TTA ^1^
*KE1* Probe	TCA TAG TTA TGC CCA TTA ^1,2^
KEB #51	TCA TAG TTA TGC CCA TTA

**^1^** The sequence represented by REB #116 contains a single mismatch (shown in bold letters) with the *KE1* probe and is observed in approximately 9.5% of the REB sequences in the database. The sequence represented by REB #115 contains two mismatches (shown in bold letters) with the *KE1* probe and is observed in approximately 90.5% of the REB sequences in the database. **^2^** The polymorphism in the middle of the *KE1* probe binding region (shown underlined) is a determinant of reactivity with the *KE1* probe.

Collectively, capture probes for a particular species detected all specimens of that species. However, amplicons from a few of the species did not react with all probes for that species. This is likely due to polymorphisms that exist within the probe binding regions. For example, recent barcoding data divided all Canadian big brown bats (BBB) (*Eptesicus fuscus*) into two distinct populations (western and eastern) [47]. A capture probe designed from available sequences of BBB from British Columbia (*BB-W*) and a probe designed to sequences of BBB from the rest of Canada (*BB-E*) were able to differentiate the two populations using Mean + 0.25SD as a cutoff for positivity (Figure 3(B)). 

It has been suggested that certain members of the *Myotis* genus, notably *M. keenii*, *M. evotis* and *M. lucifugus*, are not clearly differentiated by mitochondrial genome analyses and are accordingly often referred to as the *M. lucifugus* complex [48]. However, in a previous study of Canadian bat barcoding, specimens of these three species were clearly assigned to discrete phylogenetic clades with the exception of just one specimen that could not unequivocably be designated as *M. keenii* or *M. evotis* [29]. The present study has employed a subset of the collection examined previously so that the phylogeny of all of these samples was already known. Unfortunately the unclassified specimen was not available for inclusion in this study but interrogation of its sequence revealed that it contained no mismatches with probe *LE2*, one mismatch with *KE2* and two mismatches with both LB probes; accordingly it would most likely have been identified as *M. evotis*. Apart from this single sample, at least from a Canadian perspective, identification of these three species by barcoding and microarray analysis appears to be straightforward and a relatively large number of specimens belonging to these species have been tested in the current study. However, further COI characterization of specimens of these species throughout their ranges would be helpful in further defining the degree of inter-specific and intra-specific sequence diversity exhibited at this locus.

The PCR and microarray assay described can be used to genetically identify Canadian bat specimens to the species level using DNA extracted from small amounts of tissue from bat carcasses. This method could complement traditional taxonomic methods of species assignment especially when specimens are deteriorated, incomplete, or are of species that share highly related morphological features. Although only 14 of the 17 indigenous bat species were included in this study it is anticipated that as genetic information on the three remaining species is acquired, extra probes will be added to the microarray to allow their detection. Moreover, it is quite possible that climate change will result in the future northwards movement of bat species from the USA into Canada such that expansion of the array to enable identification of additional bat species will be required. One of the advantages of the microarray is the large number of capture probes that can be incorporated to provide additional redundancy and to increase assay resolution for differentiation of a larger number of species. This feature could potentially broaden the application of this technology to areas such as wildlife survey and conservation. Iatrogenic incursions of bats from further afield is also possible though probably highly infrequent; such specimens would likely give negative or inconclusive results with the microarray described and will likely require traditional barcoding or high density resequencing arrays for sequence characterization.

A number of prior studies have combined barcoding and microarray methods, mostly by employing traditional glass slide microarrays, to facilitate species identification. The scope of these prior studies varied widely. In one study Affymetrix technology was used to develop methods for the identification of a wide range of mammalian species [35]; another study targeted a range of European fish species [37]. Some studies have focused on more limited numbers of species restricted to specific regions and/or animal orders [34,38] while others have explored the utility of microarrays to identify species likely to transmit infectious disease agents [36,38]. While the present study has used a similar approach to design and validate a tool for a very specific purpose, further development of this technique could enlarge its applicability. One possibility is to expand the array for use in other jurisdictions by adding probes for the differentiation of a larger number of species, e.g., the 39 species of indigenous North American bats for which cases of rabies have been reported [49]. The principle could also be extended to the analysis of the much larger number of bat species that inhabit tropical regions of the Americas, many of which harbor RV variants [50]; indeed an array that identifies many neotropical bat species from Guyana based on their barcodes [8] has been described previously [35]. Alternatively, the adaptation of this method to novel far less labour intensive microarray platforms could be beneficial. For example, use of a single instrument that fully integrates and automates a complex molecular assay from nucleic acid extraction and gene amplification to array-based detection would yield a highly user-friendly microarray platform with a much more streamlined workflow. An instrument that uses an inexpensive bio-contained disposable reaction cartridge to perform such a workflow has recently been developed and applied to the detection of viruses responsible for human sexually transmitted diseases [51]. A fully automated and integrated assay for molecular species identification that does not require user handling after sample addition may be more cost-effective and rapid than DNA sequencing for applications described in this study. Thus, future work will focus on the adaptation of the methods described in this report to such highly automated platforms. As a result genetic-based tools for bat species identification will become more accessible, not only for laboratory based analysis but also for studies of bats in the field where live specimens could be rapidly and unequivocally identified using wing-punches or hair buds. 

## 4. Conclusions

A new PCR that successfully amplified the COI gene of a reference collection of 152 out of 153 bats representing 14 different species of bats that have been submitted for rabies diagnosis in Canada was developed. Amplification of COI from a highly degraded sample required an alternate primer pair. A microarray-based method for identification of chiropteran specimens to the species level was also successfully developed and validated with 153 field samples. This chiropteran PCR and microarray assay would allow unequivocal identification to species of most, if not all, bat specimens diagnosed as rabid in Canada. This new test for species assignment of rabies positive bats can be used in conjunction with existing methods for rabies virus typing to provide scientific information into the host range and host association of various rabies virus variants in bat species in Canada. The associations made between bat species and rabies virus variants will improve knowledge of rabies epidemiology and provide much needed information to support ongoing surveillance of rabies and to assess the public health risk to Canadians. Further planned expansion of this barcoding microarray assay to include more probes for redundancy and allow differentiation of additional species, and adaptation to a fully automated platform would make this technology accessible to a wider group of potential users such as those involved in wildlife conservation. 
